# Diagnostic Disparities in Erythema Visibility: A Call to Redefine Inflammatory Assessment in Diverse Skin Tones

**DOI:** 10.7759/cureus.94930

**Published:** 2025-10-19

**Authors:** Alyssa Forsyth, Stuti Prajapati, Kelly M Frasier, Charles Kriebel, Travis Jackson, Raquel Batista, Faith Jean, Udokama Ezekwe

**Affiliations:** 1 Dermatology, Texas College of Osteopathic Medicine, Fort Worth, USA; 2 Dermatology, St. John’s Episcopal Hospital, Far Rockaway, USA; 3 Dermatology, Northwell Health, New Hyde Park, USA; 4 Dermatology, Midwestern University Chicago College of Osteopathic Medicine, Downers Grove, USA; 5 Dermatology, University of Missouri School of Medicine, Columbia, USA; 6 Dermatology, Upstate University Hospital, Syracuse, USA; 7 Dermatology, Meharry Medical College, Nashville, USA; 8 Dermatology, Temple University Hospital, Philadelphia, USA

**Keywords:** erythema visibility, health care disparities, inclusive medical education, inflammatory skin disease, skin of color

## Abstract

Disparities in the diagnosis of erythema across different skin tones present a significant barrier to equitable dermatologic care. These disparities arise from a focus on lighter skin tones in clinical training and diagnostic criteria, leading to challenges in accurately identifying erythema in individuals with Fitzpatrick skin types IV to VI. Redness associated with vasodilation may not be visibly apparent or can manifest differently on darker skin, resulting in underdiagnosis or misclassification of inflammatory skin conditions. Current assessment tools, like the Investigator’s Global Assessment (IGA) and the Eczema Area and Severity Index (EASI), emphasize visible erythema and lack validated alternatives for variations in pigmentation. To promote diagnostic equity, there is a need to update inflammatory assessment methods by integrating objective metrics such as high-frequency ultrasound and infrared imaging, while also developing new training protocols that include diverse skin representations. By redefining erythema with inclusive terminology and incorporating diverse datasets in machine-learning tools, we can enhance diagnostic accuracy and ensure equitable care in inflammatory dermatology for all populations.

## Introduction and background

Inflammation of the skin is characterized by increased blood flow to superficial capillaries, resulting in a change in skin tone to a reddish hue known as erythema. Erythema is a cardinal sign, known as “rubor”, in the classic Celsus cardinal signs of inflammation, in addition to calor, dolor, tumor, and function laesa [1]. Diagnostics in dermatology primarily rely on what is visually assessed on the patient’s skin. Consequently, relying on instincts and habits when assessing human skin is prone to human error [2]. Moreover, a lack of training on the skills needed to diagnose skin of color (SOC) is evident, as clinicians report more ease and confidence in diagnosing the same condition in lighter skin tones [3]. This challenge highlights the importance of clarifying how erythema fits as a marker in clinical decision-making.

Common inflammatory skin conditions, including atopic dermatitis, psoriasis, and rosacea, depend on erythema as a primary sign to establish the diagnosis. Visual manifestations of inflammation on the skin may reveal the severity or urgency of the underlying disease process, making early detection and possible treatment an option when the need is apparent. However, this is based on the assumption that erythema will translate equally across different skin tones. Depending on erythema as a classic sign to look for may mislead clinicians when assessing patients with darker skin tones, as the redness may be subtle or masked by pigmentation [4]. In cases of obscured inflammation, with deepening skin tones, the color to look for may not be what is typically expected with erythema but instead may present as rather violaceous, gray, or brown hues [5]. This variation can result in missed or delayed diagnosis of conditions such as eczema or rosacea.

This narrative review was conducted through a targeted search of databases and search engines, including PubMed and Google Scholar, to identify peer-reviewed articles, reviews, and commentaries addressing the diagnostic limitations and disparities that arise when erythema is used as a primary indicator of inflammation across diverse skin tones. Through this synthesis, we propose a redefinition for how inflammatory skin conditions are assessed to ensure equitable dermatologic care. Prioritizing diversity in medical education may help decrease the disparities in the diagnosis of skin conditions in SOC [6]. We aim to advocate for patients by highlighting the impact of misdiagnosis using current definitions of inflammatory assessment in Fitzpatrick skin types IV-VI. We do this by promoting confidence in a clinical eye that is attuned to nuance and reflective of the diverse hues in which inflammatory skin conditions present. 

## Review

Historical and structural context

Eurocentric Roots of Dermatologic Standards

Eurocentric perspectives have long influenced dermatology as a field. As a result, skin conditions are diagnosed primarily based on how they appear in people with lighter skin tones. Medical education often emphasizes historical principles, neglecting similar conditions in individuals with darker skin tones. This inequality has led to lasting consequences for diagnosing common skin conditions. For example, the Fitzpatrick skin classification system was first developed in 1975, and initially only included lighter skin tones (I-IV) [7]. It was not until 13 years later that darker skin tones (V-VI) were added to the classification system to increase inclusivity. Despite progress in the representation of darker skin tones within dermatology, the lingering influence of Eurocentric perspectives still impacts the field. It is necessary to adopt a more inclusive approach to assessing skin conditions, particularly for individuals with darker skin. Such an approach should fully address the diverse ways in which various conditions manifest across all skin types.

Underrepresentation of SOC in Training and Literature

The lack of diverse skin tone representation in dermatology is not limited to textbooks. This lack of adequate depiction of skin conditions across all skin tones directly affects clinical training and diagnostic accuracy. Medical students and other professionals often do not receive sufficient exposure to skin conditions in patients of color. This gap is especially pronounced in textbooks and published research studies. For example, a recent study of dermatology textbooks found only 20.1% of images featured individuals with Fitzpatrick skin types IV-VI [8]. When darker skin types are underrepresented and underrecognized in training materials, it yields negative consequences, such as missed diagnoses. One study suggested medical students had lower diagnostic accuracy rates when identifying skin conditions in individuals with darker skin tones [9]. Such educational gaps can leave future clinicians unprepared for the conditions they are more likely to encounter in darker skin tones, further contributing to ongoing disparities in care.

Systemic Bias in Diagnostic Frameworks

The systemic bias in dermatology training transcends medical education. This bias tends to be incorporated into various diagnostic tools. Skin conditions can present vastly differently across individuals with varying skin tones. For example, erythema does not always appear as a "redness" on darker skin tones. Oftentimes, erythema may appear violaceous on individuals with SOC. Adhering to modern-day diagnostic tools may lead to misdiagnosis of certain conditions. Standard scoring systems such as the Eczema Area and Severity Index (EASI) rely on the extent of erythema to characterize the severity of affected areas [10]. This can skew skin assessments for individuals with darker skin tones. This bias highlights the need for updated diagnostic criteria that reflect how conditions appear across all skin tones. Fortunately, there is increasing momentum towards change. Tools beyond visual assessment are being explored as objective measures to improve diagnostic accuracy. One example is using high-frequency ultrasound to assess dermal thickness in various conditions. Overall, there is an increased need for updated diagnostic guidelines for many skin conditions, including policies that represent individuals across all skin tones.

Clinical consequences of diagnostic disparities

Erythema is a core diagnostic feature that plays a central role in racial disparities in the assessment of inflammatory skin diseases. As a primary visual marker of cutaneous inflammation, erythema is often less visible in richly pigmented skin, limiting its reliability in patients with darker skin tones [11,12]. This reduced visibility contributes to underdiagnosis and misclassification, particularly in inflammatory skin conditions such as atopic dermatitis (AD), where erythema is a heavily weighted criterion. Differences in disease distribution further compromise diagnostic accuracy; for example, AD more commonly presents on extensor rather than flexural surfaces in Black and Hispanic patients, which may be unfamiliar to clinicians [11]. These factors highlight the limitations of erythema as a universal marker of disease activity and underscore the need for more inclusive diagnostic approaches.

The clinical consequences of these diagnostic disparities are substantial, often delaying both recognition and effective treatment. Inflammatory dermatoses go unrecognized or are misclassified due to obscured erythema and may progress before appropriate interventions are initiated. This is particularly concerning in AD, which disproportionately affects racial and ethnic minority populations. Black and Hispanic individuals experience a higher prevalence of AD compared to White individuals [13], and Black children are nearly six times more likely to present with severe disease [14]. When erythema is not detected adequately, the severity of the disease may be underestimated. This can lead to inadequate treatment plans, disease progression, and poor long-term outcomes.

Beyond diagnosis and treatment, relying on erythema-centered diagnostic criteria also affects clinical research by systematically excluding patients with darker skin tones. Many commonly used scoring systems for inflammatory dermatoses, such as AD and psoriasis, prioritize erythema as a central measure of disease severity. When erythema is underrecognized in individuals with deeply pigmented skin, they may be misdiagnosed as having milder disease or excluded entirely from clinical trial eligibility. This underrepresentation limits the generalizability of trial outcomes and contributes to therapeutic inequities, as efficacy and safety data are disproportionately derived from populations with lighter skin [11,12]. Structural limitations in assessment hinder diagnostic equity and restrict access to new therapies, underscoring the need to reevaluate existing scoring systems.

Limitations of existing assessment tools

Clinician-reported outcome measures for inflammatory skin diseases, such as the Investigator’s Global Assessment (IGA), EASI, and Psoriasis Area and Severity Index (PASI), rely heavily on erythema as a primary indicator of disease activity [12]. However, erythema can be visually obscured in patients with darker skin pigmentation due to increased melanin content, limiting the accuracy of these tools in assessing disease severity [11,12]. This limitation contributes to misclassification and the systematic exclusion of patients with SOC from clinical trials that depend on erythema-based inclusion criteria.

Visual masking of erythema due to increased melanin content reduces the effectiveness of erythema-based tools for assessing conditions like atopic dermatitis and psoriasis. Inflammatory skin diseases can appear milder in patients with darker skin tones, even when the severity is comparable to or greater than that in lighter-skinned individuals. This discrepancy in diagnosis leads to disparities in recognition and treatment, particularly in AD, where the severity of the disease may be underestimated in Black and Hispanic populations [13]. These challenges are especially apparent in scoring systems like the EASI, which primarily rely on visual assessments of erythema to evaluate disease activity.

To investigate these limitations, Zhao et al. (2017) conducted a study on AD outcome measures in patients with different skin pigmentation, using a Mexameter to categorize participants by a melanin index [15]. Those with an index above 200 were classified as having SOC. The study assessed the reliability of traditional scoring tools like the EASI and IGA. While these tools had good overall inter-rater reliability, their performance was slightly lower in SOC patients. Notably, substituting the erythema component of the EASI with a greyscale modification maintained strong reliability (ICC = 0.78), suggesting erythema did not significantly impact assessment variability. Additionally, there was no significant difference in score variability between erythema and other EASI components in SOC patients (p = 0.47), although erythema was more consistent in lighter skin (p = 0.001) [15]. These findings indicate that outcome measures could be improved by de-emphasizing erythema. Although EASI remains the primary core measure across all skin types, its erythema-dependent components and their prominence in clinical trial frameworks can inadvertently exclude or misrepresent SOC patients. This limits the demographic inclusivity of study populations, reduces the generalizability of trial outcomes, and perpetuates disparities in dermatologic evidence and care [11,15]. Despite increased awareness of erythema's limitations across diverse skin tones, few validated alternatives have been developed or widely accepted to bridge this diagnostic gap.

Yet, even with increasing recognition of these limitations, standardized, pigment-inclusive tools designed to address skin tone variability remain largely absent from routine clinical and research practice. Tools such as EASI and PASI have not been systematically recalibrated or modified to ensure accuracy across all skin types. While exploratory approaches like greyscale modifications show potential, they remain underutilized and have not been integrated into core outcome sets or clinical trial protocols. The absence of pigment-inclusive standards hinders equitable assessment and reinforces existing disparities in dermatologic care. Addressing this gap requires developing and adopting assessment tools that are objective and adaptable to all skin tones.

Objective and non-visual diagnostic innovations

The limitations of erythema-based assessment tools in individuals with richly pigmented skin underscore the pressing need for objective, pigment-independent diagnostic strategies in dermatology. Traditional evaluation methods often rely on visual indicators, particularly erythema, which may not be readily apparent across all skin tones (Table 1). This can lead to diagnostic inaccuracies, delayed treatment, and inequitable care. In fact, in one study, participants demonstrated higher diagnostic accuracy when evaluating lighter skin (72.1%) compared with SOC (52.8%; p ≤ 0.001) [3]. In response, recent research has focused on the development of non-visual technologies that offer standardized, quantifiable measures of cutaneous inflammation. Approaches such as high-frequency ultrasound, infrared thermography, and spectrophotometric analysis are promising for improving diagnostic accuracy while minimizing dependence on subjective visual assessment.

**Table 1:** Variability of Erythema Presentation Across Skin Types in Common Inflammatory Dermatoses Fitzpatrick skin types I–III (types that burn easily and tan minimally to moderately) and IV–VI (types that burn minimally and tan easily to deeply) are used to stratify skin responses to UV exposure, which can influence erythema visibility and diagnostic assessment in inflammatory skin conditions. EASI: Eczema Area and Severity Index

Condition	Erythema Appearance in Light Skin (I–III)	Erythema Appearance in Dark Skin (IV–VI)	Clinical Considerations
Atopic Dermatitis (AD)	Bright red, easily detected	Violaceous, gray, or brown; may be subtle	AD distribution may differ; severity underestimated using EASI’s erythema component [14,15]
Psoriasis	Red plaques with scaling	Violaceous-brown plaques; scale may dominate	Visual erythema underrepresented; palpation and texture assessment recommended [12]
Rosacea	Diffuse facial redness	Subtle hyperpigmentation; redness may be masked	Focus on flushing, telangiectasias, or edema rather than redness alone [4,5]
Lupus Erythematosus	Erythematous malar rash	Violaceous, hypopigmented, or dusky; more severe	Consider biopsy or UV photography if diagnosis uncertain [16]
Drug Eruption	Diffuse erythema	Dusky or hyperpigmented patches	Observe for edema, systemic involvement; visual assessment alone may be insufficient [4]

High-frequency ultrasound (HFUS) is a modality that evaluates inflammatory skin diseases. HFUS provides reproducible, objective insights into clinical and subclinical inflammation by measuring dermal and epidermal thickness. This is especially valuable in situations where it is hard to visually detect erythema. Iyengar et al. (2018) showed that HFUS correlates well with disease activity in AD by detecting epidermal thickening [17]. Similarly, Niu et al. (2021) confirmed HFUS’s efficiency in characterizing dermal swelling and inflammatory infiltrates [18]. Likewise, Etesami et al. (2025) showed that ultrasound combined with shear wave elastography (SWE) can distinguish active from inactive morphea lesions with high sensitivity and specificity, quantifying inflammatory activity in a pigment-independent manner [19]. This method assesses disease severity without relying on pigmentation.

Infrared thermography offers an additional non-invasive tool for assessing cutaneous vascular activity and perfusion. This technique captures heat patterns associated with inflammation and vasodilation, serving as an indirect yet quantifiable proxy for erythema. Ranosz-Janicka et al. (2019) reported the efficacy of infrared imaging in identifying inflammatory activity even when visual signs were equivocal [20]. Szczepanek et al. (2022) highlighted the potential of infrared imaging to visualize vascular changes that may otherwise be missed in patients with darker skin [21]. While thermography assesses vascular changes through heat signatures, spectrophotometric and colorimetric methods offer complementary approaches.

Spectrophotometric and colorimetric technologies also provide objective metrics by quantifying skin chromophores such as melanin and hemoglobin. These tools reduce observer bias by analyzing light absorption and reflectance. These devices eliminate subjectivity by measuring light absorption and reflectance, standardizing skin assessments. Szczepanek et al. (2022) demonstrated that colorimetric analysis can reliably detect erythema irrespective of baseline pigmentation [21]. Earlier work by Stamatas and Kollias (2007) validated diffuse reflectance spectroscopy to differentiate between melanin and hemoglobin signals [22]. This supports its application in evaluating inflammation and pigmentation with high sensitivity and consistency.

These non-visual diagnostic methods represent a step forward in providing more inclusive dermatologic care. Technologies such as HFUS, infrared thermography, and spectrophotometric analysis can bridge diagnostic disparities through reliable, pigment-independent measures of disease activity.

Bias in technology and artificial intelligence (AI)

Underrepresentation in Training Datasets

Before reconstructing and improving equitable dermatologic care for people with SOC, deeply rooted technological biases should be identified. Future healthcare leaders are receiving incomplete training tools, thus hindering optimal diagnosis and treatment of erythematous conditions. Underrepresentation of how erythema presents in diverse skin tones should be avoided, deconstructed, and revised. For example, the representation of patients with SOC is limited in influential, published images in medical journals and online resources [16]. Proficient and inclusive skin care requires that dermatologists are equipped with adequate resources and knowledge of how diseases are represented on all skin types, including darker skin tones. Limited awareness in recognizing skin conditions in darker skin types contributes to these collective disparities and ultimately delays care [23]. It may be helpful to provide diverse educational images and health data at each step along the training pathway. Underrepresentation of SOC should be addressed at the basic science level and reinforced by diverse, inclusive material [24]. Preparing current and future healthcare leaders with comprehensive skills might better afford equitable dermatologic care across racially diverse skin tones.

Algorithmic Risk of Perpetuating Visual Bias

Dermatologists are responsible for providing not only comprehensive and inclusive care, but also care in a timely manner. It would be helpful if technological tools incorporated pigmentary diversity to avoid perpetuating visual biases. The development of inclusive diagnosis systems comprising diverse images that represent the broad range of skin tones could aid in earlier diagnoses and treatment [25]. These systems can improve the treatment of inflammatory disorders and skin cancers in patients with SOC. While automated triage systems provide many positive support modalities to dermatologists, there is still a risk of misdiagnosing complex skin disorders on SOC. It may be helpful to provide thorough algorithmic diagnostic aids encompassing diverse skin tones. For example, erythema in darker skin tones presents with reduced blanching reactions, requiring multicultural perspectives to effectively treat [26]. With pigmentary nuance integrated into diagnostic aids, we can ensure cultural competence in dermatologic healthcare providers. Evidence suggests medical students exhibited less diagnostic accuracy in identifying urticaria, squamous cell carcinoma, and atopic dermatitis in darker skin tones [27]. Actively opposing the perpetuation of visual bias is the responsibility of each healthcare provider, from medical students to attending physicians.

Design Recommendations for Inclusive AI Diagnostics

AI can be a powerful supplemental tool in the dermatology sphere and the greater healthcare world. Just as humans are constantly learning and rewiring neural circuitry, continuous adjustments should be made to AI designs to ensure they are nuanced and inclusive of all skin tones. The Monk Skin Tone Scale has recently been added to AI databases to better assess the broad spectrum of skin tones [28]. This scale can be used in conjunction with Fitzpatrick Skin Types in creating inclusive AI algorithms. The process of reducing AI biases and diagnostic disparities is complex and constantly evolving. This can be seen in variability and inconsistency with baseline performances across computer-based systems [29]. This evidence highlights the importance of healthcare provider competency when diagnosing and treating dermatologic conditions in conjunction with AI tools. To increase consistency and reproducibility, it has been proposed to create an international collaboration to provide diverse images for AI programs [30]. The complex but worthwhile implementation of AI enhancement may better prepare future dermatologists with the knowledge to treat diverse skin tones. 

Educational reform and redefinition of erythema 

Updating Curricula and Diagnostic Terminology

Improving educational resources and using more descriptors can help identify erythema amongst Fitzpatrick skin types. A lack of experience and exposure can lead to misdiagnosis or underdiagnosis due to the failure to detect erythema. Dodd et al. reported that medical students have less diagnostic accuracy for conditions like shingles and Lyme disease and less confidence in identifying several common clinical presentations, such as urticaria and chickenpox, in non-White participants compared to White participants (p < 0.01 for both) [31]. This demonstrates that current students do not receive enough exposure to disease presentations in various skin tones to accurately identify them. These findings illustrate a growing need for more exposure, such as through access to specially curated databases of SOC images, clinical rotations in hospitals and clinics serving diverse populations, and grand rounds on SOC cases if accessibility is an obstacle. Moreover, the current diagnostic term of “erythema” is a limiting factor. When redness is absent, it is commonly assumed that erythema is absent. This thought process effectively rules out many differentials when a patient presents. Due to the concentration of melanin in darker skin tones, redness appears more violaceous, gray, or brown. Acknowledgment of this spectrum is key to providing adequate and accurate care to patients with darker skin tones.

Incorporating Diverse Skin Images and Examples 

The underrepresentation of dark skin tones in medical textbooks and journals continues to be a pressing issue within dermatology. In a 2006 study, it was reported that major dermatology textbooks consisted of images with dark skin tones ranging from 4-19% [32]. This implies limited exposure to SOC in academic training at the time, contributing to uncertainty in identifying and recognizing presentations such as erythema in diverse skin types. A 2020 study revealed similar percentages of SOC images in dermatology textbooks to the 2006 study, despite technological advancements in photography, illustrating the stagnant nature of the curricula [33]. A focused effort to photograph and present disease processes and presentations, side by side with the lighter skin tones, can improve recognition in medical personnel. In the context of erythema in SOC, highlighting the varying appearances, such as violaceous, gray, dark brown, hyperpigmentation, or hypopigmentation, is important to improving recognition and, thus, a more accurate diagnosis.

Deconstructing the Color-Centric Concept of Erythema

Erythema, derived from erythros in Greek, means “redness”. This definition dominates the dermatological space, failing to account for variations in different Fitzpatrick skin types. However, the focus on this specific color could prove harmful by delaying diagnosis. Finlay et al. highlight how the definition of erythema has been misconstrued to describe skin changes in all skin types, making the use of the term null [4]. Erythema is often used to mean inflammation. However, while erythema is a sign of inflammation, it is not necessarily inflammation in itself. This implies that describing skin changes at face value and using more literal descriptors may be more effective in determining diagnosis. In darker skin types, other signs of inflammation, such as calor (redness), tumor (swelling), and dolor (pain), could serve as supplemental identifiers of erythema. These could be examined via high-frequency ultrasound, infrared imaging, and spectrophotometric analysis. In more ambiguous cases where inflammation may not be the cause, a biopsy could be obtained to gain a better clinical picture. Focusing on associations and causes of erythema, rather than the visualization of redness when evaluating SOC, may provide more accurate assessments.

Ethical, equity, and policy implications

Diagnostic Equity as a Healthcare Justice Issue

An imperative ethical component when providing unbiased dermatological care is justice. Alongside the other principles of medical ethics, distributive justice emphasizes that all patients receive equal treatment [34]. How can people with SOC be treated equally if there is less training and exposure to erythematous pigmentary nuances? This question demonstrates the cyclical nature of both the conflicts and the barriers preventing equitable dermatologic treatment. Image-based learning datasets used to educate dermatologic providers are supplied in various modalities, from textbooks to AI programs. It is important to comprehensively note and monitor flaws in the system. For example, there are disproportionate representations of several phenotypic variables in anatomy textbooks [35]. As newer, more inclusive textbooks are printed, each healthcare provider has a responsibility to address these barriers and make the necessary changes to provide more informed and inclusive diagnoses. With a duty to provide fair and equal healthcare, it may be helpful for dermatologists and all other providers to lean on collaboration and team support. For example, diversity in dermatology residency programs and sharing of information might reduce gaps in patient care and improve diagnostic equity [36]. By maximizing preparation and support, dermatologists can potentially provide both equal and timely care to patients with all types of skin tones. 

Promoting Inclusivity in Clinical Research and Treatment Eligibility

There should be inclusivity of a broad spectrum of skin tones in clinical dermatologic research to achieve optimal, equitable care. Both diversity and transparency in clinical trial parameters and methods should be emphasized. While there have been improvements in policies, studies still lack reporting and consistent diversity in clinical trials [37]. Inclusive and diverse clinical research may enhance equitable treatment of richly pigmented skin tones. To redefine how erythematous skin conditions are approached, changes should be made in the academic and clinical dermatologic research domains. Racial diversity barriers can be hidden and less identifiable to medical professionals. This emphasizes the importance of being aware that previous research and datasets prioritize lighter skin phenotypes. With this awareness, clinical and research dermatologists can take into account a person’s skin type, race, and ethnicity, thus providing culturally competent care [38]. Barriers of inadequate training on how erythema presents differently on diverse skin tones should not impact the appropriate course of treatment. These barriers might be present in the way disease severity is defined. One study found that moderate to severe facial erythema in patients with SOC was not visible in clinical grading [39]. The underrecognized racial barriers in clinical research and treatment eligibility can be deconstructed using a proactive mindset to emphasize cultural competence and inclusivity. 

Policy Strategies for Standardizing Inclusive Practices

An essential component of breaking down the deeply rooted barriers of classical erythema definition is creating a standard treatment process. While this is an expansive and constantly evolving criterion, baseline policies should strive to equally diagnose and treat inflammatory skin conditions across diverse populations. Checkpoints to remind busy providers to be aware of biases might be a strong, standard tool in medical policies. For example, Harvard University has previously created implicit bias tests to challenge everyone to recognize their biases [40]. Support and transparency in an open and fair forum might allow each dermatology provider to assess implicit biases, leading to more equitable treatment. A promising step in standardizing inclusive practices and reducing misdiagnoses is the newer generations of dermatoscopes with cross-polarized filters. Cross-polarized dermatoscopes are extremely useful in providing accurate clinical assessments of patients with SOC who heal with hyperpigmentation [41]. An attempt to standardize cultural competence and the inclusion of tools and accountability are important steps in providing equal care. Integration and influence from diversity organizations should also be emphasized in medical settings. Organizations like the Skin of Color Society (SOCS) promote education and awareness of issues related to SOC racial disparities [42]. These steps might aid in establishing timely and equal care for differently presenting erythematous skin conditions. Each healthcare provider is responsible for remaining active, conscious, and honest in reducing perpetual biases and, above all, optimizing patient health outcomes.

## Conclusions

There is a significant disparity in diagnosing erythema across different skin tones. Key issues include an overreliance on erythema for diagnosis, insufficient training for SOC, biases in diagnostic tools, and a lack of objective metrics. These challenges hinder equitable dermatological care for individuals with darker skin types, leading to underdiagnosis, delayed treatment, and exclusion from research. To address these gaps, medical education must be updated to help trainees identify erythematous conditions in darker skin, even when typical redness is not visible. Diagnostic equity should be recognized as a healthcare justice issue. Dermatologists and other healthcare providers must pursue ongoing education on SOC, promote inclusivity in research, and adopt culturally competent practices. Addressing this disparity is crucial for reducing racial and ethnic inequalities in dermatology and healthcare overall, ultimately improving health outcomes for patients of color.
